# Can Prenatal Malaria Exposure Produce an Immune Tolerant Phenotype?: A Prospective Birth Cohort Study in Kenya

**DOI:** 10.1371/journal.pmed.1000116

**Published:** 2009-07-28

**Authors:** Indu Malhotra, Arlene Dent, Peter Mungai, Alex Wamachi, John H. Ouma, David L. Narum, Eric Muchiri, Daniel J. Tisch, Christopher L. King

**Affiliations:** 1 Center for Global Health and Diseases, Case Western Reserve University, Cleveland, Ohio, United States of America; 2 Division of Vector Borne Diseases, Nairobi, Kenya; 3 Kenya Medical Research Institute, Nairobi, Kenya; 4 Malaria Vaccine Development Unit, National Institute of Allergy and Infectious Diseases, Bethesda, Maryland, United States of America; 5 Veterans Affairs Medical Center, Cleveland, Ohio, United States of America; Rigshospitalet, Denmark

## Abstract

**Background:**

Malaria in pregnancy can expose the fetus to malaria-infected erythrocytes or their soluble products, thereby stimulating T and B cell immune responses to malaria blood stage antigens. We hypothesized that fetal immune priming, or malaria exposure in the absence of priming (putative tolerance), affects the child's susceptibility to subsequent malaria infections.

**Methods and Findings:**

We conducted a prospective birth cohort study of 586 newborns residing in a malaria-holoendemic area of Kenya who were examined biannually to age 3 years for malaria infection, and whose malaria-specific cellular and humoral immune responses were assessed. Newborns were classified as (i) sensitized (and thus exposed), as demonstrated by IFNγ, IL-2, IL-13, and/or IL-5 production by cord blood mononuclear cells (CBMCs) to malaria blood stage antigens, indicative of in utero priming (*n* = 246), (ii) exposed not sensitized (mother *Plasmodium falciparum* [Pf]+ and no CBMC production of IFNγ, IL-2, IL-13, and/or IL-5, *n* = 120), or (iii) not exposed (mother Pf−, no CBMC reactivity, *n* = 220). Exposed not sensitized children had evidence for prenatal immune experience demonstrated by increased IL-10 production and partial reversal of malaria antigen-specific hyporesponsiveness with IL-2+IL-15, indicative of immune tolerance. Relative risk data showed that the putatively tolerant children had a 1.61 (95% confidence interval [CI] 1.10–2.43; *p* = 0.024) and 1.34 (95% CI 0.95–1.87; *p* = 0.097) greater risk for malaria infection based on light microscopy (LM) or PCR diagnosis, respectively, compared to the not-exposed group, and a 1.41 (95%CI 0.97–2.07, *p* = 0.074) and 1.39 (95%CI 0.99–2.07, *p* = 0.053) greater risk of infection based on LM or PCR diagnosis, respectively, compared to the sensitized group. Putatively tolerant children had an average of 0.5 g/dl lower hemoglobin levels (*p* = 0.01) compared to the other two groups. Exposed not sensitized children also had 2- to 3-fold lower frequency of malaria antigen-driven IFNγ and/or IL-2 production (*p*<0.001) and higher IL-10 release (*p*<0.001) at 6-month follow-ups, when compared to sensitized and not-exposed children. Malaria blood stage–specific IgG antibody levels were similar among the three groups.

**Conclusions:**

These results show that a subset of children exposed to malaria in utero acquire a tolerant phenotype to blood-stage antigens that persists into childhood and is associated with an increased susceptibility to malaria infection and anemia. This finding could have important implications for malaria vaccination of children residing in endemic areas.

*Please see later in the article for Editors' Summary*

## Abstract 2

In a prospective cohort study of newborns residing in a malaria holoendemic area of Kenya, Christopher King and colleagues find a subset of children born to malaria-infected women who acquire a tolerant phenotype, which persists into childhood and is associated with increased susceptibility to malarial infection and anemia.

## Editors' Summary

**Background:**

Each year, *Plasmodium falciparum*, a mosquito-borne parasite, causes about 500 million cases of malaria and about one million people die as a result. Most of these deaths occur in young children in sub-Saharan Africa. Indeed, malaria accounts for a fifth of all childhood deaths in Africa, which makes it one of the most important childhood infectious diseases in this region. Very young children—those up to 6 months old—are relatively resistant to high-density parasitaemia and to clinical malaria, but children between 6 and 36 months old have an increased susceptibility to parasitaemia and to clinical malaria. Parasitaemia is the presence of *P. falciparum* parasites in the blood; a high density of blood-stage parasites causes the symptoms of clinical malaria (including high fever) and life-threatening organ damage and anemia (a lack of red blood cells).

**Why Was This Study Done?:**

The age-dependent pattern of susceptibility to malaria suggests that young babies are protected by antibodies provided by their mothers, but that by 6 months old, when these antibodies have largely disappeared, babies have not yet fully developed their own anti-malaria immunity. However, little is known about the acquisition of anti-malaria immunity in infants, a process that needs to be understood in order to design effective vaccines for this age group. In particular, it is unclear how maternal malaria infection affects the acquisition of anti-malaria immunity. Malaria in pregnancy may expose the unborn child to malaria-infected red blood cells and to soluble malaria antigens (molecules that the immune system recognizes as foreign). This exposure could increase or decrease the child's immune responses to blood-stage malaria antigens and thus affect his/her ability to fight off malaria. In this study, the researchers investigated how prenatal malaria exposure affects anti-malaria immunity in young children and their susceptibility to subsequent malaria infections.

**What Did the Researchers Do and Find?:**

The researchers determined which of 586 newborn babies enrolled into their study in an area of Kenya where malaria is very common had been exposed to *P. falciparum* before birth by looking for parasites in their mother's blood at delivery. They looked for malaria-specific immune responses in T cells (a type of immune system cell) in the newborn babies' cord blood by measuring the production of cytokines (molecules that either activate or inhibit the immune system) by these cells after exposure to malaria antigens. Finally, they examined the infants twice yearly for 3 years for malaria infection, malaria-specific immune responses, and anemia. The researchers classified the babies into three groups; cord blood cells of “sensitized” babies made activating cytokines in response to malaria antigens; cord blood cells of “exposed, not-sensitized” babies did not make activating cytokines but made an inhibitory cytokine (IL-10); and “not-exposed” babies were born to mothers with no *P. falciparum* infection at delivery. In their first 3 years of life, the exposed, not-sensitized group had a 60% greater risk of malaria infection (measured by counting parasites in their blood) than the unexposed group and a slightly higher risk of malaria infection than the sensitized group. They also had lower hemoglobulin levels (a sign of anemia) than the other babies. At age 6 months, the T cells of exposed, not-sensitized children were less likely to make activating cytokines in response to malaria antigens but made more IL-10 than the T cells of the other children; malaria-specific antibody levels were similar in the three groups.

**What Do These Findings Mean?:**

These findings suggest that some children who are exposed to malaria before birth become “tolerant” to blood-stage malaria antigens. Exposure to malaria antigens before birth “tricks” their T cells into recognizing these antigens as self antigens. This immune tolerance, which persists into childhood, reduces the ability of the immune system to attack and destroy parasites and increases the susceptibility of these tolerant children to malaria infection. Why some children who are exposed to malaria before birth become tolerant while exposure to malaria antigens “primes” the immune system of other children to respond efficiently to these antigens is not clear. However, these findings could have important implications for the design of malaria vaccines for use in areas where children are often exposed to malaria before birth and for the design of strategies for the prevention of malaria during pregnancy.

**Additional Information:**

Please access these Web sites via the online version of this summary at http://dx.doi.org/10.1371/journal.pmed.1000116.

- This study is further discussed in a *PLoS Medicine* Perspective by Lars Hviid
- Information is available from the World Health Organization on malaria (in several languages)
- The US Centers for Disease Control and Prevention provides information on malaria (in English and Spanish)
- Information is available from the Roll Back Malaria Partnership on all aspects of global malaria control, including information on malaria in pregnancy and on children and malaria
- MedlinePlus provides links to additional information on malaria (in English and Spanish)

## Introduction

Falciparum malaria is one of the most important pediatric infectious diseases in sub-Saharan Africa, where it is estimated to kill at least 1 million children per year. In areas where malaria transmission is stable, infants and children typically experience clinical morbidity and high blood stage parasite densities. Infants up to 6 months old appear to be relatively resistant to clinical malaria and high-density parasitemia, while children between 6 and 36 months of age appear to have increased susceptibility to these clinical and parasitological outcomes [1]. Although evidence supporting a direct role for passively acquired maternal IgG antibodies in mediating protection against infant malaria is inconsistent [2], epidemiologic studies show that malaria susceptibility increases after maternal antibodies have waned but before immunity to blood-stage *Plasmodium falciparum* (Pf) can develop due to repeated infections [3]. Knowledge of the immune mechanisms underlying changes in infant malaria susceptibility may have important implications, not only for the fundamental understanding of human malaria immunobiology, but also for evaluation of the efficacy of future malaria vaccine initiatives with this age group.

The physiologic and immunologic effects of maternal malaria infection on the fetus complicate the acquisition of anti-malaria immunity in children. Pf-infected erythrocytes have the propensity to sequester in the intervillous blood of the placenta, a condition referred to as placental malaria. Placental malaria is most common in women with their first or second pregnancy, seemingly due to the lack of acquired immunity to Pf clones that preferentially bind to the placental vasculature [4],[5]. In this context, the fetus may be exposed to soluble malaria antigens or infected erythrocytes that gain access to the fetal circulation after crossing the placenta. Although it is not feasible to document this process in vivo, indirect evidence supporting its occurrence includes the demonstration of T and B cell responses to crude schizont extracts and blood-stage antigens in cord blood lymphocytes, a cell population that represents circulating fetal lymphocytes present at the time of birth [6]–[9]. The consequences of this in utero immune experience on infant malaria immunity are not known, particularly whether it accelerates the development of protective antibodies or whether cellular immune responses are inhibited by virtue of sensitization and/or subsequent immune tolerance mechanisms.

The significance of fetal malaria experience is underscored by epidemiologic studies suggesting that offspring of women with placental malaria are more susceptible to malaria during infancy than those of mothers without placental malaria [10]–[12]. These studies, however, do not explore the mechanisms for this increased susceptibility. Although the presence of placental malaria or malaria during pregnancy is associated with fetal priming to malaria blood stage antigens [7]–[9],[13],[14], some offspring fail to acquire protective responses in the presence of maternal malaria, suggesting the fetus may acquire immune tolerance [15],[16]. Offspring of women with malaria at delivery, for example, have evidence for impaired acquisition of malaria-specific antibodies [17], as well as reduced levels of functional antibodies that inhibit invasion of erythrocytes by the membrane-anchored MSP1_19_, a protein required for entry of the parasite into red blood cells [18]. It is unknown whether prenatal exposure to malaria results in persistent expansion or suppression of malaria blood stage–specific cellular or humoral immunity, and whether this affects infant susceptibility to malaria infection and disease.

We describe here the results of a prospective cohort study of infants born in a malaria-holoendemic area of Kenya. The study was designed to test the hypothesis that prenatal malaria experience, reflected by maternal malaria infection at parturition and neonatal cord blood T cell sensitization to several malaria blood stage invasion ligands, could influence susceptibility to malaria infection and risk of anemia in the first 3 years of life. To understand the mechanism associated with the altered risk for malaria, we measured whether this prenatal experience influences acquisition of cellular and humoral immune responses to these same invasion ligands, as measured biannually in children during the first 3 years of life. Merozoite surface protein 1 (MSP1) is the most abundant merozoite protein and a leading malaria vaccine candidate [19],[20]. PfP0 is also a merozoite surface protein, antibodies to which block Pf erythrocyte invasion in vitro [21],[22]. Apical membrane antigen 1 (AMA1) and the F-2 region of the 175 kDa erythrocyte binding antigen (EBA-175) are proteins released from rhoptries or micronemes, organelles within the merozoite, following initial merozoite attachment to erythrocytes [23]–[25]. These proteins were studied because they represent important vaccine candidates to which artificially or naturally acquired antibodies have been shown to block Pf erythrocyte invasion in vitro; in non-human primates, immunization with them produces partial immunity to blood-stage infection [25]–[27].

## Methods

### Study Participants and Study Design

Approval for the study was obtained from the Kenya Medical Research Institute National Ethical Review Committee and the Institutional Review Board for Human Studies at Case Western Reserve University. Mothers provided witnessed written informed consent for participation and assent for their infants. All women included in the study were screened for HIV as part of a volunteer counseling and testing (VCT) program supported by the International Center for Reproductive Health and the Kenyan Ministry of Health starting in 2002. Counseling and perinatal treatment with nevaripine was provided through this program. All HIV results were kept strictly confidential. For women recruited before 2002 who had not participated in the VCT program, and following written informed consent, stored frozen plasma samples (at −80°C) from mothers were examined by HIV-ELISA as described [28]. For offspring of women who tested positive for HIV, testing for the virus was performed on frozen samples that had been collected in follow-up visits with their mothers.

Pregnant women were recruited from the antenatal clinic at Msambweni District Hospital, Kwale District in Coast Province, Kenya. They underwent a detailed questionnaire that queried their education level, spouse's occupation, and household income. Malaria transmission in this area is stable, with seasonal variation related to rainfall [29]. Women enrolled in the study were given malaria prophylaxis consisting of single-dose sulfadoxine–pyrimethamine (SP) at the beginning of the second and third trimesters of pregnancy, in accordance with recommendations from the Kenya Ministry of Health. Paired maternal venous blood, placental intervillous blood, and umbilical cord blood from the newborns were collected at the time of birth. Cord blood was collected as described [15] and the absence of placental alkaline phosphatase (PLAP) indicated the sample did not have significant contamination by maternal blood [30]. Infant venous blood samples (∼5 ml) were collected beginning at 6 mo of age and every 6 mo thereafter until age 36 mo. Plasma was stored at −80°C until antibody assays were performed. Cellular immune responses at birth and during subsequent follow-ups were performed on fresh cells.

### Malaria Infection Status

Maternal venous blood, intervillous placental blood, and cord blood were examined for malaria infection status by two methods. In the LM method, thick and thin blood smears were prepared, fixed in methanol, stained with Giemsa, and examined by light microscopy (LM) for Pf-infected erythrocytes in 100 high-powered fields. The density of parasitemia was expressed as the number of asexual *P. falciparum* per microliter of blood, assuming a leukocyte count of 8,000/µl. In the PCR method, DNA was extracted from 200 µl of whole intervillous blood and the red cell pellet of anticoagulated maternal and cord blood (Qiagen). Real-time quantitative PCR (RTQPCR) was performed, with 2.5 µl of DNA used as a template for amplification of the multicopy Pf 18S small subunit ribosomal RNA gene [31],[32]. The increased sensitivity of the RTQPCR assay in comparison to LM analysis for Pf malaria has been described in detail [31]. A newborn was considered “exposed” to malaria in utero if one or more of the blood smear preparations or RTQPCR results from the various compartments were positive, and/or CBMC had a recall response to malaria blood stage antigens. A newborn was considered to be “not exposed” when both diagnostic tests were negative for all three blood compartments and CBMC lacked a recall response to malaria blood stage antigen.

### Helminth Infections and Hemoglobin

Stool and urine were obtained at delivery and examined for the presence of intestinal helminths and schistosome ova, respectively, as described [33]. Night blood samples were obtained from pregnant women prior to delivery, and 1 ml of heparinized blood filtered for the presence of *Wuchereria bancrofti* microfilariae as described [34]. *W. bancrofti* was also assessed by a circulating antigen assay using stored frozen plasma samples collected at delivery, based on the Og4C3 antigen detection assay (TropBioMed). Samples were obtained during follow-up periods if participants were unable to provide samples at delivery. Women infected with intestinal helminths or schistosomes were treated with mebendazole (500 mg in a single dose) or praziquantel (40 mg/kg in a divided dose), respectively, according to the Kenyan Ministry of Health recommendations after delivery. No specific therapy was given for *W. bancrofti* infections, because a district-wide treatment program was started during the study (October 2002) as part of the WHO-sponsored global program to eliminate filariasis. Hemoglobin levels were assessed from fresh peripheral blood samples in children using the HemoCue system (HemoCue).

### Antigens, Mitogens, and Recombinant Cytokines

For the T cell studies in cord blood and peripheral venous blood, both peptides and recombinant antigens were used. For MSP1, two peptides were used that correspond to well-characterized T cell epitopes within the C-terminal 83 kDa fragment of MSP1 (GYRKPLDNIKDNVGKMEDYIKK, codons 250–271; KLNSLNNPHNVLQNFSVFFNK, codons 101–121) [15]. Three peptides were used corresponding to N- and C-terminal regions of PfP0, denoted N1 (DNVGSNQMASVRKSLR; codon 33–48), N2 (SVRKSLRGKATILMGKNT; codon 42–59), and C1 (AKADEPKKEEAKKVE; codon 285–299) as previously described [35]. The peptides were synthesized and purified to >95% (ResGen) and subjected to analysis by MHC Class-II Binding Peptide Prediction algorithms (Propred and Tepitope). They were found to have broad binding specificity to many MHC class II alleles including DRB1*0401, DRB1*0101, and DRB1*1501, which are common in Kenya [36]. Recombinant EBA175 (codons 144–753) corresponding to region II (the binding domain to sialic acid on erythrocytes [23]) was expressed in *Pichia* [37]. The MSP1_42_ was expressed in *E. coli* and properly refolded as described [38]. Tetanus toxoid was obtained from the Massachusetts Department of Health and used at a dilution of 1∶200. Phorbol 12-myristate 13-acetate (PMA) plus ionomycin (Calbiochem) were used as positive mitogen controls in parallel cultures for cytokine assays. For lymphocyte proliferation assays performed on a subset of CBMC, the mitogen control was phytohemagglutinin (PHA, Sigma) at 5 µg/ml.

### Cord and Peripheral Blood Lymphocyte Cultures

Cord blood was obtained from the umbilical vein immediately after delivery and anticoagulated with heparin. Similarly, maternal peripheral venous blood was obtained by venipucture and anticoagulated with heparin. CBMCs and peripheral blood mononuclear cells (PBMCs) were isolated from the freshly obtained blood by density gradient centrifugation on Ficoll-Hypaque as described [15]. CBMCs were cultured at a density of 2×10^6^/ml in a total volume of 0.5 ml in duplicate in 48-well flat-bottomed plates (Costar), in RPMI-1640 supplemented with heat-inactivated 10% autologous plasma, 4 mM L-glutamine, 25 mM HEPES, and 80 µg/ml gentamicin (Biowhittaker). For lymphocyte proliferation studies, CBMCs were cultured at 2×10^6^/ml in a total volume of 200 µl in triplicate.

In preliminary studies, heat-inactivated autologous plasma was not suppressive when compared to serum-free medium in activated lymphocyte cultures (unpublished data). By contrast, we occasionally observed high background and nonspecific lymphocyte reactivity using fetal calf serum or pooled human sera (unpublished data). For PBMC cultures 10% fetal calf serum was used instead of autologous plasma (Hyclone; with low reactivity). Lymphocyte cultures were also performed at a density of 1×10^6^/ml in a total volume of 200 µl in triplicate for both cytokine production and lymphocyte proliferation assays. For lymphocyte proliferation studies 1 µCi of ^3^H-thymidine (New England Nuclear) was added to each culture and harvested 16 h later as described [39]. ^3^H-thymidine was measured with a beta-counter (Packard Matrix).

For CBMCs the following culture conditions were used: medium alone (negative control); 10 µg/ml MSP1 P2 and P3 peptides; 10 µg/ml PfP0 N1, N2, or C1 peptide; 5 µg/ml EBA175; and PMA (50 pg/ml) plus ionomycin (1 µg/ml) in duplicate. CBMCs were cultured with individual peptides except when there were insufficient cells and MSP1 and PfP0 peptides were pooled. For PBMCs, MSP1 and PfP0 peptides were pooled for all participants, because of limited blood volume. In a subset of children with larger lymphocyte recoveries, peptides were cultured individually. Pooling peptides in CBMC or PBMC cultures did not affect the levels of antigen-induced cytokine production or lymphocyte proliferation (unpublished data). All culture supernatants were immediately frozen at −80°C following culture.

In a subset of CBMCs (i.e., cord blood collected from all deliveries September through November 2002), recombinant human IL-2 and IL-15 (10 U/ml, BD Biosciences) were added to parallel cultures, with medium alone or with malaria antigens, to assess the effect of these cytokines on reversing immune hyporesponsiveness. Lymphocyte proliferation responses, with addition of rIL-2+rIL-15 and malaria antigens, divided by lymphocytes cultured with rIL-2+rIL-15 alone (i.e., malaria antigen+rIL-2+rIL-15/rIL-2+rIL-15), were calculated, and we designated a positive response when this ratio exceeded 2.

### Measurement of Cytokines and Criteria for a Positive Response to Malaria Antigens by CBMCs

Quantification of IFNγ, IL-5, IL-13, IL-2, and IL-10 was performed on culture supernatants collected at 72 h by ELISA [15]. Results were expressed in pg/ml by interpolation from standard curves based on recombinant lymphokines [40]. Antibody pairs for capture and detection (all biotinylated) for the cytokines studied were as follows: IL-5, 18051D and 18522D (BD-Pharmingen); IFNγ, M-700A and M-701B (Endogen); IL-13, P-130-E and M-130-B (Endogen); IL-2, MAB-602 and BAF-202 (R&D Systems); IL-10, 18551D and 18652D (BD-Pharmingen). A positive response was scored when both of the following criteria were fulfilled: (1) a net value for antigen-stimulated cells at least 2-fold greater than that of parallel cultures containing media alone. If cytokine production was not detectable in the negative control cultures, then >40 pg/ml cytokine was considered to be a positive response; and (2) responses to two or more malaria antigens.

IFNγ ELISPOT for both CBMCs and PBMCs was also performed at 48 h as described for all samples [41]. A positive response was scored when one of the following conditions were met: (1) ≥4 IFNγ-secreting cells/4×10^5^ CBMCs or PBMCs in response to at least two malaria antigens when no IFNγ-secreting cells were present in negative control wells (media alone); (2) in cases where IFNγ-secreting cells were observed in negative control wells, the number of spots generated by antigen-driven cord blood lymphocytes (CBLs) was at least 2-fold greater. Based on these criteria, no malaria antigen-driven IFNγ-secreting cells were detected in CBMC from 16 healthy North American newborns or PBMCs from ten malaria-naïve adults.

### Measurement of IgG in Plasma to MSP1_42_, AMA1, and EBA175-Region II

IgG antibodies to recombinant Pf MSP1_42_ (3D7 and FVO alleles) and AMA1 (3D7 and FVO alleles) and region II EBA175 were measured by ELISA as described previously [42],[43]. Briefly, Immunolon 4 plates were coated with 0.1 µg/ml MSP1_42_ or AMA1, and 0.2 µg/ml of EBA175 in PBS. Plates were subsequently blocked with 3% bovine albumin in PBS (Sigma) to which 1∶200 final dilution of cord or peripheral blood plasma was added and incubated at 4°C overnight. Plates were subsequently washed, and a 1∶2,000 dilution of goat anti-human IgG conjugated alkaline phosphatase added and incubated for 2 h at 37°C (H&L recognition, Jackson ImmunoResearch). A pool of ten cord blood plasma samples with high antibody reactivity was used as a standard, with a 1∶200 dilution used as high standard and given the arbitrary value of 100. The pooled plasma was subsequently serially 2-fold diluted eight times to generate the standard curve. A positive control plasma sample with IgG antibodies that recognized all recombinant antigens tested was also added to each plate. Plasma from nine North American adults never exposed to malaria was used as the negative control. Positive values were greater than the mean+3 standard deviations (SDs) of the value of the individual negative control plasma samples.

### Statistics

All statistical analysis was performed using SAS version 9.1. Statistical comparisons of continuous variables according to prenatal malaria exposure were performed using the Mann-Whitney test, Kruskal Wallis test, or Student's *t*-test of log-transformed values, depending on data distribution and number of exposure categories analyzed. Categorical outcomes were analyzed using the Chi-square test. Repeated measures of categorical outcomes (i.e., infection status, cytokine outcomes, and antibody responses) were analyzed with generalized estimating equations [44] using the PROC GENMOD procedure with a binomial distribution for the response variable with a logit link function. Repeated continuous outcomes (i.e., parasite density and hemoglobin) were analyzed with repeated measure models using the PROC MIXED procedure, as previously described [45]. These methods allowed us to accommodate repeat observations among the same children. Time was included as a linear variable and its interaction term with prenatal malaria exposure category was evaluated for rate of change in outcomes over time. Parasite counts were log-transformed to normalize the data. All models included the dichotomous covariates of season and study site. The auto-regressive covariance structure was used for both sets of repeated measure analyses as this structure gave the best fit based on Akaike's information criterion, the corrected Akaike's information criterion, and the Bayesian information criterion as provided in the SAS 9.1 output.

## Results

### Study Population and Classification of Prenatal Immune Status

**Figure 1:**
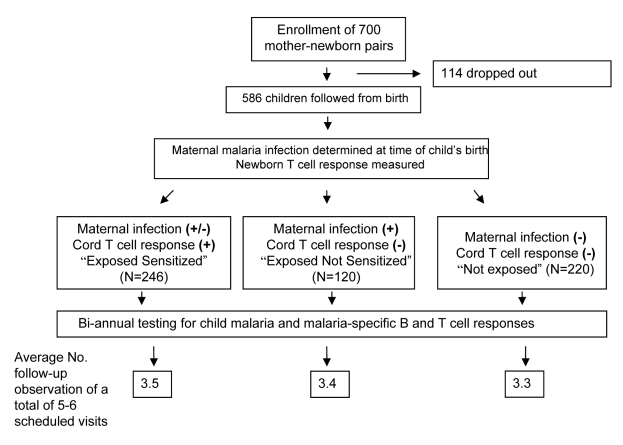
Flow diagram of birth cohort study.

An overview of the enrollment and follow-up of the participants is presented in Figure 1. A total of 700 mother–newborn pairs were recruited between 2000 and 2003 with 586 children (83%) enrolled in the birth cohort. One hundred fourteen children were excluded because of insufficient CBMC collection, non-viable CBMCs (no response to mitogens), or failure to return for any follow-up visits. (The major reason for failure to return for any visits was emigration from the study area). The average number of follow-up visits was 3.5 out of 5.3 potential visits, or 66% follow-up. Children recruited late in the study did not have a full 3 y of follow-up.

Newborns were categorized as “exposed not sensitized,” “exposed sensitized,” and “not exposed” groups (Table 1). Exposed not sensitized infants were defined as offspring of malaria-infected pregnant women, but lacking evidence for fetal priming. Exposed sensitized infants were defined as showing cytokine production to malaria-blood stage antigens by CBMCs as described in Methods. Mothers of all but 43 sensitized newborns had malaria at delivery. Lymphocyte proliferation was not measured in all CBMCs and therefore not used as an indicator of fetal priming. Malaria antigen–driven IL-10 production alone was not considered a criterion for fetal priming because non-T cells can generate IL-10. The not-exposed group included offspring of pregnant women who had no evidence of malaria, and who did not have detectable priming to malaria antigens in cord blood.

**Table 1:** Demographic characteristics of maternal–fetal pairs according to newborn malaria exposure status and cord blood T cell responses to MSP1, PfP0, and EBA-175.

Newborn Category	Exposed Not Sensitized	Exposed Sensitized	Not Exposed	*p*-Value
Maternal/newborn infection with malaria^a^	Yes	Yes/no^b^	No	—
Newborn T cell response to malaria antigens	No	Yes	No	—
Number of children	120	246	220	—
Males/females	60/60	133/113	117/103	0.50^c^, 0.65^d^
Average age of mother at delivery (y)	25.3	25.9	26.4	0.61^c^, 0.19^d^
Primigravida/multigravida mothers	39/80 (49%)	66/178 (37%)	53/165 (32%)	0.10^c^, *0.02* d
Weeks of first antenatal clinic visit prior to delivery (average, range)	5.3 (1–20)	4.1 (1–18)	4.5 (1–16)	0.16^c^, 0.30^d^
Reported bednet use in home	13%	15%	17%	0.88^c^, 0.58^d^
HIV-positive women (*n* = 464 tested)	13/99 (13.1%)	17/200 (8.5%)	9/165 (5.5%)	0.22^c^, *0.04* d
HIV-positive offspring	4/13	4/17	3/9	0.70^c^, 1.0^d^
Proportion of women with schistosomiasis	11/89 (12.3%)	24/214 (11.2%)	26/169 (15.3%)	0.84^c^, 0.58^d^
Proportion of women with lymphatic filariasis	31/80 (38.8%)	79/163 (48.5%)	77/138 (55.8%)	0.17^c^, *0.02* d
Proportion of women with hookworm	37/62 (60.0%)	91/143 (63.6%)	55/137 (40.1%)	0.64^c^, *0.01* d

a Maternal/newborn Pf infection was assessed by either LM and/or RTQPCR detection of parasites in maternal, cord, and/or intervillous placental blood.

b Forty-three of the individuals in this group had T cell responses, but without maternal/newborn malaria infection at delivery.

c *p*-Value of exposed not sensitized compared to exposed sensitized group.

d *p*-Value of exposed not sensitized compared to not exposed group.

The demographic characteristics of the three groups are also shown in Table 1. Mothers of newborns who were “exposed” to malaria at birth (sensitized and not sensitized) were slightly younger than mothers of newborns who were “not exposed”. Almost half of the exposed not sensitized children (49%) had primigravid mothers, a proportion significantly greater than with mothers of not-exposed offspring (32%). Although women and their newborns were enrolled just prior to delivery, antenatal clinic records were available for many women. Women presented for their first antenatal clinical visit on the average of 4–5 wk before delivery, which was similar between the study groups. According to Kenyan Ministry of Health Guidelines, a single prophylactic dose of SP should be administered at the beginning of the second and third trimester and not less than one month before expected date of delivery. Since we did not record whether women received SP or not, we cannot state with certainty whether there was a difference in malaria prophylaxis between groups. The observation, however, that the approximate gestational age at first antenatal clinic attendance between the groups was similar suggests that the amount of malaria chemoprophylaxis between groups is unlikely to be markedly different. The proportion of women that reported bed net use was similar between the study groups (Table 1). The socioeconomic status, as determined by highest education level attained by both parents, household income, and type of parental employment, was similar with respect to exposed not sensitized children compared to the sensitized group (*p* = 0.23, *p* = 0.11, and *p* = 0.37 respectively) and not-exposed group (*p* = 0.54, *p* = 0.091, and *p* = 0.28 respectively).

The proportion of mothers with HIV or helminth coinfections is also shown in Table 1. Overall 8.4% (*n* = 39) of 464 mothers tested were HIV positive. Mothers of the exposed not sensitized group had higher HIV positivity than offspring in the not exposed group. HIV was transmitted to 11 offspring (28%), with a similar proportion of HIV positive offspring in each group (Table 1). The portion of helminth-infected women was similar among the three groups except that mothers of the not exposed group were more likely to be infected with lymphatic filariasis, but had lower rates of hookworm infections.

### Exposed Not Sensitized Newborns Have Evidence of T Cell Anergy and Increased IL-10 Production Indicative of Immune Tolerance

**Figure 2:**
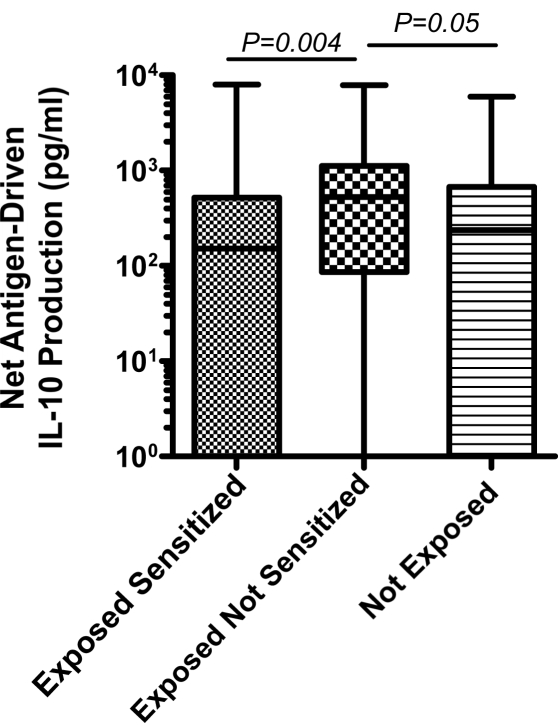
Median malaria antigen-driven IL-10 production for all subjects in the three experimental groups.

**Figure 3:**
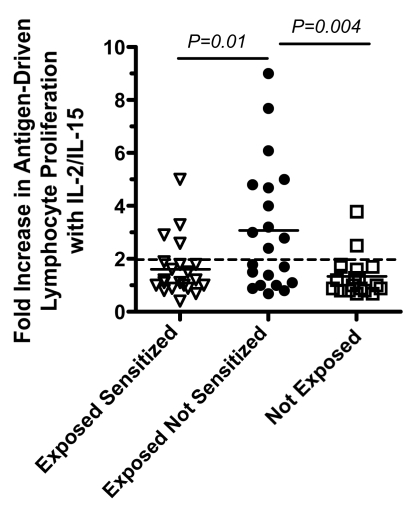
Augmentation of malaria-antigen driven lymphocyte proliferation by CBMCs in a subset of exposed sensitized (*n* = 21), exposed not sensitized (*n* = 21), and not exposed (*n* = 19) newborns in the presence of IL-2+IL-15. Values shown are the fold-increase in cpm in cultures with IL-2+IL-15+malaria antigens divided by cultures with IL-2+IL-15 alone. A positive response was considered >2-fold increase (dashed horizontal line). Statistical differences were calculated by Chi-square comparing the proportion of positive responders. Solid horizontal bars indicate median values.

To examine whether active suppression may contribute to the lack of detectable malaria blood stage antigen driven IFNγ, IL-2, IL-5, and/or IL-13 response in the exposed not sensitized group, malaria antigen driven IL-10 production by CBMCs was also examined in culture supernatants. IL-10 production in the exposed not sensitized group was higher than in the other groups (Figure 2). To determine whether malaria-specific anergy may also contribute to the lack of T cell responses among the exposed not sensitized newborns, cord blood from a subset of newborns (*n* = 61) was cultured in the presence of IL-2+IL-15 and malaria antigens and compared to cultures with IL-2+IL-15 in the absence of malaria antigens. In 21 of the exposed not sensitized children that previously lacked detectable lymphocyte proliferation responses or cytokine production, 11 (52%) had detectable antigen-specific proliferative responses in the presence of IL-2+IL-15. By contrast, malaria antigen with IL-2+IL-15 augmented four of 21 (19%) in the exposed sensitized group and two of 17 (11%) in the not-exposed children (Figure 3). This indicates the presence of lymphocyte anergy in the exposed not sensitized group and suggests immune tolerance.

### Putatively Tolerant Offspring Are More Susceptible to Malaria

During the follow-up period, malaria infection assessed by LM was detected at least once in 42.9% (51/119) of exposed not sensitized (putatively tolerant) children, 35.0% (86/246) of exposed sensitized children, and 27.2% (58/217) of not-exposed children (*p* = 0.012). At least one malaria infection assessed by PCR was observed in 67.2% (80/119), 64.2% (158/246), and 55.7% (121/217) of these children, respectively (*p* = 0.066). Since malaria transmission can vary over time and location, and to adjust for repeated measures on children, time, and location, we used a generalized estimating equation to model infection status (Table 2). Exposed not sensitized children had a 61% greater risk of infection compared to not exposed children and 41% greater risk compared to sensitized children. Similar trends were found for PCR diagnosis: Exposed, not sensitized children had a 39% greater risk of infection over time compared to sensitized children and 33% greater risk compared to not exposed children.

**Table 2:** Association of prenatal immune experience with susceptibility to *P. falciparum* infection in early childhood.

Group	*P. falciparum* Infection Frequency^a^		Longitudinal Analysis Accommodating Repeated Measures^b^: Relative Risk of Exposed Not Sensitized to Other Groups	
	LM	PCR	LM, Relative Risk (95% CI)	PCR, Relative Risk (95% CI)
Exposed, not sensitized	0.213	0.412	—	—
Exposed, sensitized	0.175	0.363	1.41 (0.97–2.07); *p* = 0.074	1.39 (0.99–1.98); *p* = 0.053
Not exposed	0.123	0.317	1.61 (1.10–2.43); *p* = *0.024*	1.33 (0.95–1.89); *p* = 0.097

a Values indicate the population mean of the ratio of a positive result to total observations for each individual. The average number of observations for each group is shown in Figure 1.

b Generalized estimating equation adjusted for location, time, and repeated measures as described more fully in the Methods section.

When parasite density was analyzed using a repeated measures model, putatively tolerant children acquired an extra 1.30 parasites/µl (95%CI 1.01–1.68, *p* = 0.043) or 1.78 parasites/µl (95%CI 1.05–3.01, *p* = 0.032), compared to sensitized children, assessed by LM or RTQPCR respectively every 6 mo, adjusted for season and location. Parasitemia was similar among infected individuals in the exposed sensitized, exposed not sensitized and not-exposed groups, respectively (geometric mean parasitemia of 531, 643, and 437 parasites/µl by blood smear [*p* = 0.77] and 770, 998, and 953 parasites/µl by RTQPCR [*p* = 0.93]), indicating that differences in the frequency of malaria infection account for much of the difference in parasitemia between groups. Of note, when parity was entered in the analysis, it was not associated with child susceptibility to infection or parasitemia.

### Putatively Tolerant Children Are at Greater Risk of Anemia

**Figure 4:**
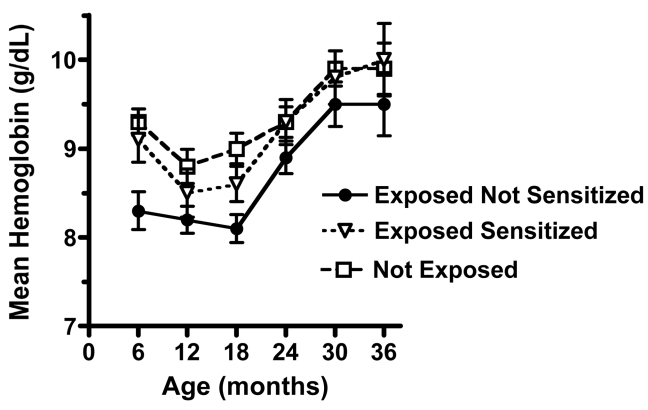
Exposed not sensitized children at birth show reduced mean hemoglobin levels throughout 36 months of follow-up. The overall mean hemoglobin levels in exposed not sensitized (putatively tolerant) children were significantly lower than the exposed sensitized (*p* = 0.03) and not exposed children (*p* = 0.01) based on a repeated measures model (described in Table 2). Analysis was adjusted for children's height and weight (BMI) and helminth coinfection.

To determine whether prenatal experience influenced the risk of anemia, hemoglobin levels were measured over the period of follow-up for offspring that were sensitized, putatively tolerant or not exposed (Figure 4). Hemoglobin level for all children initially declined with age then increased by 30–36 mo. Mean hemoglobin levels in the exposed not sensitized group were consistently lower than in the other two groups (Figure 4). To test whether hemoglobin levels were significantly different between the groups we used a mixed linear model that adjusts for repeated measures over the period of observation and also adjusts for coinfections with schistosomiasis, lymphatic filariasis and/or hookworm, as well as height and weight measurements (body mass index [BMI], a surrogate for nutritional status) from each follow-up. The mean (±SD) adjusted hemoglobin levels for the putatively tolerant group were 8.6±0.24 g/dl compared to the sensitized group (9.1±0.19 g/dl, *p* = 0.03) or the unexposed group (9.2±0.15 g/dl, *p* = 0.01).

### The Relationship of Fetal Immune Experience with Malaria-Specific B and T Responses in Childhood

**Figure 5:**
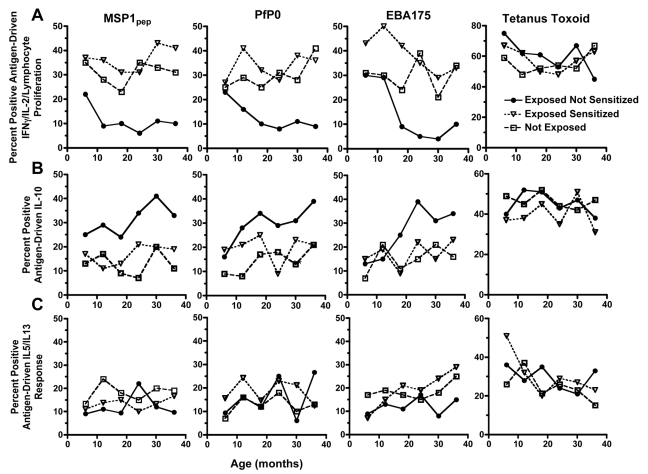
The association of prenatal malaria exposure with the frequency of malaria antigen and tetanus toxoid-driven cytokine production measured biannually to 36 months of age. (A) Exposed not sensitized children have a lower frequency of IFNγ and IL-2 production, and/or lymphocyte proliferation by PBMCs (a positive response is defined in the text and method section) compared to exposed sensitized children and not-exposed children using a generalized estimating equations that adjusts for time, location, and repeated measures as more fully described in the Methods section (relative risk, [95% confidence interval], exposed not sensitized children compared to exposed sensitized children: 0.28 [0.15–0.37], *p* = 0.0003 for MSP1; 0.33 [0.20–0.51], *p* = 0.0008 for PfP0; 0.36 [0.19–0.74], *p* = 0.005 for EBA175; and 1.07 [0.89–1.65] for tetanus toxoid). Similar effect sizes were observed for exposed not sensitized children compared to not exposed children (unpublished data). (B) The frequency of antigen-driven IL-10 is higher in the exposed not sensitized children than in exposed sensitized (relative risk for MSP1, 1.67 [1.35–2.73], *p* = 0.0007; for PfP0 1.45 [1.03–2.51], *p* = 0.02; for EBA175 1.38 [0.99–2.42], *p* = 0.05; and for tetanus toxoid 1.04 [0.85–1.12], *p* = 0.73. (C) The frequency of malaria antigen-induced IL-5 and/or IL-13 cytokine responses are equivalent for the three groups (relative risk for exposed not sensitized compared to exposed sensitized children: for MSP1, 0.97 [0.79–1.21], *p* = 0.35; for PfP0 0.92 [0.73–1.36], *p* = 0.47; for EBA175, 0.83 [0.64–1.09], *p* = 0.16; and for tetanus toxoid, 1.01 [0.93–1.06], *p* = 0.61. Similar effect sizes were observed for exposed not sensitized children compared to not exposed children (unpublished data).

In order to understand the immunologic basis for the increased susceptibility of putatively tolerant offspring to malaria, we examined how prenatal exposure would affect the child's subsequent acquisition of cellular and humoral immune responses. A positive cellular response was defined as one or more cytokines and/or lymphocyte proliferation response to the malaria antigen or peptide, as indicated in the Methods section. Using a repeated measures model, we observed 64% to 72% lower proportion of exposed not sensitized (putatively tolerant) children produced IFNγ or IL-2, and/or showed lymphocyte proliferation to MSP1, PfP0, and EBA175 by PBMCs, compared to sensitized children during the period of follow-up (Figure 5A). These impaired antigen-specific Th1-type responses in putatively tolerant children were malaria-specific, since tetanus toxoid (TT)-driven IFNγ, IL-2, and/or lymphocyte proliferation responses were comparable between the three groups. Generally, if a child failed to generate a Th1-type recall response to malaria blood stage antigens at one time during infancy, they also failed to do so during other follow-up assessments. By contrast, 38% to 67% higher proportion of putatively tolerant children produced more antigen-driven IL-10 during the period of follow-up compared to the other two groups (Figure 5B). There was no difference in the proportion of children's PBMCs that produced IL-5 and/or IL-13 to MSP1, PfP0, and/or EBA175 between the three groups (Figure 5C). In summary, putatively tolerant newborns have an impaired malaria-specific Th1-type cytokine response, a reciprocal increase in antigen-driven IL-10 production, and a similar Th2-type cytokine release, when compared to other children, and these differences persist through early childhood.

**Figure 6:**
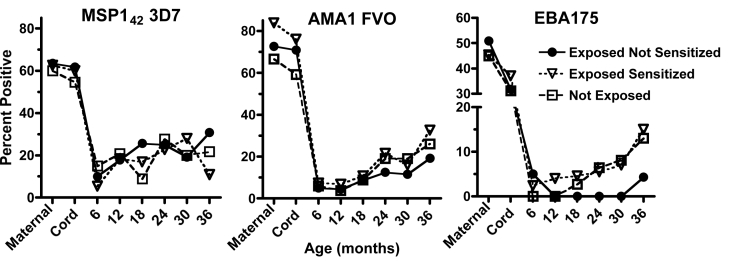
The association of prenatal exposure to malaria with acquisition of MSP1_42_ and AMA1, and EBA-175 specific IgG responses in all children. There were no differences between the proportion of children with antibody responses to MSP1_42_ and AMA1 between the three groups beginning at 12 months of age as determined by a generalized estimating equation described in Figure 5 (exposed not sensitized group compared to exposed sensitized group: relative risk for MSP1_42_, 1.05 [0.88–1.17], *p* = 0.457; for AMA1, 0.87 [0.74–1.11], *p* = 0.251), but a significant reduction for EBA-175–specific IgG, 0.41 [0.28–0.97], *p* = 0.03. Similar effect sizes were observed for exposed not sensitized children compared to not exposed children (unpublished data).

There was no difference in the proportion (nor levels, unpublished data) of children that acquired IgG antibody responses to MSP1_42_, or AMA1 with increasing age between the three study groups as determined from 12 mo of age (Figure 6). Both alleles (3D7 and FVO) for MSP1_42_ and AMA1 were tested and showed similar age-related patterns of response. By contrast, tolerant children failed to acquire any antibody response to EBA175 until 3 y of age (Figure 6). Antibody responses to PfP0 were not measured, as properly folded recombinant protein was unavailable. Notable was the high proportion of mothers with antibodies to each of the recombinant proteins at delivery in which the antibodies were successfully transferred to their offspring. Of note, antibody levels dropped dramatically by 6 mo of age and then slowly increased over time (Figure 6). Generally if a mother or child had an antibody response to one recombinant antigen, they had a response to the other allele or recombinant antigens measured, although this was not always the case.

## Discussion

Results from the current study support the hypothesis that fetal malaria experience contributes to regulation of childhood malaria immunity in endemic areas where infection during pregnancy is common. We first described a subset of children whose mothers are infected with malaria in late pregnancy, but whose cord blood lymphocytes obtained at delivery failed to produce common T cell cytokines to several malaria blood stage antigens. We speculate that these children are likely to be exposed to malaria blood stage antigens in utero, but generate an immune phenotype that we refer to as putatively tolerant. This immune tolerant phenotype is based on the observation that approximately 90% of these children consistently failed to generate specific malaria blood stage antigen–induced Th1-type cytokine responses, compared with approximately 50% of other children, even though the risk of infection tended to be higher in the immune tolerant group. Further, these immune tolerant children showed increased malaria antigen–driven IL-10 production by cord blood lymphocytes that persisted through childhood, suggesting the presence of T regulatory cells specific for malaria blood stage antigens [46]. Although the current study did not show whether the malaria antigen–driven IL-10 originated from T cells, we and others have previously shown that malaria blood stage antigens stimulate IL-10 production by CD4^+^ T cells in CBMCs from malaria-exposed newborns and in PBMCs from children, but not in cells from newborns in North America [15],[47].

Further evidence for prenatal immune experience in the putatively tolerant group is that antigen-specific proliferation responses can be detected in over half of a randomly selected subset of tolerant newborns following the addition of IL-2 and IL-15, indicative of lymphocyte anergy [48],[49]. The mechanisms for immune hyporesponsiveness in this tolerant subset of children are likely to be complex and involve multiple pathways. For example, we have identified a population of malaria-specific CD4^+^CD25^bright^FoxP3^+^ cells in cord blood of malaria infected women that can suppress antigen-driven CD4^+^CD25^dim^ cells (Mackroth, et al., personal communication). Brustoski and colleagues [46] also found increased IL-10 production by CBMCs in response to malaria blood stage antigens, and they noted that depleting CD4+CD25^bright^ cells abrogates parasite-specific IL-10 production and increases IFNγ-specific responses, suggesting a population of IL-10–secreting T regulatory cells in newborns. Alternatively, there may be clonal deletion of malaria-specific lymphocytes, as previously demonstrated in offspring of malaria-infected mice [50] and in analogous studies of maternal exposure to human filariasis [51].

An important consequence of this antenatally acquired immune “tolerance” is increased risk for malaria infection during childhood. Prior studies have demonstrated that offspring of women with placental malaria and/or malaria infection at delivery are more likely to be infected with malaria [10],[12]. However, the basis for this increased risk has not been well defined and the antenatal exposure to malaria not evaluated. The current study is novel in that it takes an immunological approach to better define antenatal experience with malaria and its effect on the infant's susceptibility to malaria.

A potential mechanism for this increased risk became apparent when offspring of infected women were divided into groups of children exposed to malaria in utero, in which immune priming to malaria antigens resulted, and children potentially exposed to malaria in utero but where evidence for immune priming by CBMCs was lacking (termed putatively tolerant). Tolerant children had a 61% and 34% higher incidence of malaria based on LM and PCR, respectively, using a generalized estimating equation that accommodates the individual correlation among repeated malaria measurements, as compared to not exposed children [52]. This difference is unlikely a consequence of differences in exposure, since adjustments for location and time of season did not affect the association. Thus putatively tolerant children appear to be more susceptible to malaria infection compared to not exposed children. This increased susceptibility may arise from prenatally acquired Th1-type immune hyporesponsiveness that persists into childhood. There was also a strong trend for putatively tolerant children to be more susceptible to malaria compared to the exposed sensitized children, although this relationship did not reach statistical significance. This weaker association may have resulted from misclassification of some sensitized children who may have had more than one regulatory phenotype. The fetus is likely to acquire a spectrum of immune responses to malaria blood stage antigens during pregnancy, ranging from robust Th1-type immune responses to fully immune regulatory phenotypes. Where the fetus resides on this spectrum may depend on the type and amount of malaria antigen exposure and when exposure occurs during gestation. Thus the division between these two groups is not precise. Ultimately we aim to identify one or more immune markers that better characterize an immune regulatory phenotype, such that it can be used as a continuous variable to predict susceptibility to malaria infection and disease.

There was a stronger association with susceptibility to malaria infection among the putatively tolerant group as measured by LM compared to PCR diagnosis. This is not surprising since PCR can detect more infections with low-level parasitemia, ones less likely to result in overt clinical disease.

There are several potential mechanisms that may increase susceptibility to malaria among tolerant children. The failure of 90% or more of tolerant children to acquire antigen-specific T cells that produce a Th1-type cytokine profile during the first 3 years of life may result in inadequate T cell support for antibody production and/or antibody-dependent cell mediated immunity. Humoral immunity among tolerant children, however, was similar to that observed in the other groups, as measured by the overall proportion (and levels) of malaria-specific IgG directed toward MSP1_42_ and AMA1. It is possible that these measures do not provide a good correlate of protective immunity, and more restricted antibody responses may be involved. In this context, immune tolerant children failed to acquire antibodies to EBA175 compared to other children, although the overall antibody response to EBA175 was lower than to the other antigens. We have also previously observed that tolerant children in the same population had impaired functional antibody responses to MSP1_19_ [16]. T cell help for antibody production may be intact among putatively tolerant children, as indicated by the presence of a frequency of Th2-type cytokine production by PBMCs similar to that of other children. T cell–dependent effector mechanisms, however, may be impaired. Parasite killing is augmented when antibodies act in cooperation with monocytes [53]–[55] or other FcγR-bearing cells [56]. Optimal activation of monocytes or other FcγR-bearing cells requires T cell help through release of cytokines such as IFNγ and IL-2, which was diminished in PBMCs of tolerant children during the follow-up period.

Putatively tolerant children were also at increased risk for developing anemia, showing mean hemoglobin of 0.5 g/dl lower during the follow-up period than children in the other groups. Previous studies have shown that babies born to anemic mothers (associated with malaria in pregnancy) are more susceptible to anemia (perhaps due to reduced iron stores [57],[58]). Indeed, almost half of the women in putatively tolerant group were primigravid compared to about a third in the other groups. Unfortunately, reliable hemoglobin measurements were not obtained in women at delivery, so we could not associate maternal anemia with risk of anemia during childhood. Another contributing factor to greater anemia in the putatively tolerant group is increased frequency of malaria infection, since the association with lower hemoglobin levels is reduced if the analysis is adjusted for malaria infection. This association was unlikely to be confounded by co-infections with hookworm, schistosomiasis, and/or lymphatic filariasis, since these infections are uncommon (<5% in children 3 years and younger in this population [34]). Poor nutritional status is also an important risk factor for anemia. Overall children in the cohort had good nutritional status. The BMI for all children in the cohort fell above the third percentile at each biannual follow-up visit, and most BMIs were within±1 SD of the mean based on the recently published WHO standards for developing countries [59]. The analysis was also adjusted for BMI using the linear mixed model. The severity of anemia can be influenced by the presence of sickle cell trait (HbAS) or alpha-thalassemia. These alleles are present in coastal populations of Kenyans at gene frequencies of approximately 0.13 and 0.14, respectively [60]. Individuals in the population were not genotyped, but there is no a priori reason that these genotypes should be skewed among the prenatal exposure groups.

Why some offspring of malaria infected women acquire a tolerant phenotype and others become primed to malaria antigens cannot be directly evaluated; there are technological and ethical limitations to understanding precisely when during gestation, or for how long before parturition, the fetal innate or adaptive immune response might be affected by exposure to maternal malaria. Consistent with the concept that in utero exposure to malaria is linked with placental sequestration of infected erythrocytes, we noted that a greater proportion of women whose offspring were deemed tolerant were primigravida mothers (49%), relative to mothers with sensitized offspring (37%) and unexposed women and newborns (32%). This observation suggests that the amount or duration of malarial antigen exposure, or time of exposure during gestation, may determine whether a fetus becomes tolerant or sensitized. It is possible that some tolerant newborns may not have been exposed to malaria antigens during gestation, even though the mother was infected. This possibility is hard to exclude, although our preliminary data suggest that MSP1 is commonly found in cord blood from infected women, often as immune complexes ([31] and unpublished data). Indeed, these immune complexes may facilitate the transplacental transport of malarial antigens (May, et al., personal communication), similar to transplacental antigen transport demonstrated for tetanus toxoid [61] and nonhuman insulin [62]. The types of malarial antigens that cross the placenta may also determine whether tolerance or priming occurs [63]. Fetal antigen-presenting cells (APCs) are not fully mature and provide weak costimulation to T cells compared to adult APCs [64]. If the fetus is exposed only to soluble proteins, tolerance may be induced in the form of T cell anergy, as indicated in the current study. However, if the fetus is also exposed to molecules that induce strong innate immune responses (such as parasite DNA, which we have detected in about 10% of cord blood samples [31]), this could sufficiently activate fetal APCs to prime T cells.

There are several limitations in this study. First, children were only followed biannually for the presence of malaria infection and hemoglobin levels, so malaria infections occurring between sampling times would be missed. Second, mothers were only assessed for malaria at delivery and placental histopathology was not available. Uninfected women at delivery may have been infected earlier during pregnancy. This would have misclassified some putatively tolerant children as not exposed. The effect on the study would be to reduce the observed differences between the tolerant and not-exposed groups. Some children died during the first year after birth and were not included in the cohort. We were able to ascertain seven deaths, but could not determine whether these children died of malaria or not. This could introduce a bias into the results. Finally, the presence of clinical malaria in children was not examined. This omission reduces our ability to assess whether fetal immune tolerance protects against symptomatic malaria by modulating the host inflammatory response. This possibility is suggested by reports of asymptomatic malaria in children over 6 months of age that had been closely followed since birth [40]. Studies are currently underway to address these limitations.

Once the significance of fetal malaria experience is better understood, it should translate into more effective strategies for malaria chemoprophylaxis during pregnancy. Malaria chemoprophylaxis that completely prevents infection during pregnancy, for example, may reduce fetal exposure, thereby preventing the development of immune tolerance. If this reduces both the risk of infection and disease in offspring, then such treatment would be desirable. However, if fetal immune tolerance reduces the risk of clinical disease, then prenatal chemoprophylaxis should be targeted to limiting disease and not infection per se, and linked with intermittent presumptive antimalarial chemoprophylaxis during infancy.

## Abbreviations

APC: antigen-presenting cell
BMI: body mass index
CBL: cord blood lymphocyte
CBMC: cord blood mononuclear cell
LM: light microscopy
PBMC: peripheral blood mononuclear cell
Pf: *Plasmodium falciparum*
RTQPCR: real-time quantitative PCR
SP: sulfadoxine–pyrimethamine
